# Spray Deposition of Ag Nanowire–Graphene Oxide Hybrid Electrodes for Flexible Polymer–Dispersed Liquid Crystal Displays

**DOI:** 10.3390/ma11112231

**Published:** 2018-11-09

**Authors:** Yumi Choi, Chang Su Kim, Sungjin Jo

**Affiliations:** 1 School of Architectural, Civil, Environmental, and Energy Engineering, Kyungpook National University, Daegu 41566, Korea; yums1026@gmail.com; 2 Advanced Functional Thin Films Department, Korea Institute of Materials Science (KIMS), Changwon 51508, Korea; cskim1025@kims.re.kr

**Keywords:** silver nanowire, graphene oxide, polymer-dispersed liquid crystal, smart window, hybrid transparent conductive electrode

## Abstract

We investigated the effect of different spray-coating parameters on the electro-optical properties of Ag nanowires (NWs). Highly transparent and conductive Ag NW–graphene oxide (GO) hybrid electrodes were fabricated by using the spray-coating technique. The Ag NW percolation network was modified with GO and this led to a reduced sheet resistance of the Ag NW–GO electrode as the result of a decrease in the inter-nanowire contact resistance. Although electrical conductivity and optical transmittance of the Ag NW electrodes have a trade-off relationship, Ag NW–GO hybrid electrodes exhibited significantly improved sheet resistance and slightly decreased transmittance compared to Ag NW electrodes. Ag NW–GO hybrid electrodes were integrated into smart windows based on polymer-dispersed liquid crystals (PDLCs) for the first time. Experimental results showed that the electro-optical properties of the PDLCs based on Ag NW–GO electrodes were superior when compared to those of PDLCs based on only Ag NW electrodes. This study revealed that the hybrid Ag NW–GO electrode is a promising material for manufacturing the large-area flexible indium tin oxide (ITO)-free PDLCs.

## 1. Introduction

Smart windows are controllable windows whose optical properties can be altered by applying an electric field. They are used for various applications including switchable privacy glasses, vehicle windows, and energy-saving windows [1,2,3]. Smart windows have recently attracted significant attention since they can minimize heating and cooling energies in buildings and transportation systems. Among the different electrooptically switchable active components available for smart windows such as polymer-dispersed liquid crystals (PDLCs), chromic materials, and suspended particles, PDLCs are an excellent candidate due to their high transmittance, wide viewing angle, high switching speed, and a relatively simple fabrication process [4,5].

The PDLCs consist of birefringent liquid crystal (LC) droplets that are uniformly dispersed in a solid polymer matrix. In order to fabricate smart windows based on PDLCs, the PDLC film is positioned between two transparent conductive electrodes (TCEs). The PDLC film can be switched from an opaque to a transparent state since the electric field between the TCEs aligns the directors of the LCs along the same direction. Therefore, TCEs with low sheet resistances and high transmittances are necessary in order to minimize the voltage drop across the electrode and ensure fast switching as well as smaller power consumption. Indium tin oxide (ITO) has been commonly used as a TCE for typical smart windows based on PDLCs. Although ITO has high conductivity and transmittance, its relatively high cost and fragile characteristics make it unsuitable for fabrication of large-area flexible smart windows.

There are several potential alternatives to ITO including conductive polymers, graphene, Ag nanowires (NWs), metal grids, and carbon nanotubes. Recently, conductive polymers, graphene, and Ag NWs have been successfully integrated into PDLC-based smart windows [6,7,8]. Although these emerging candidate materials are of primary interest, they suffer from lower electrical conductivities, complex fabrication processes, lower transmittances, and uneven distributions of the electric currents. In order to overcome the disadvantages of these individual TCE materials, hybrid TCEs such as Ag NW–graphene, Ag NW–conductive polymer, Ag NW–metal oxide, Ag NW–carbon nanotubes, and Ag NW–metal grids [9,10,11,12,13] have also been investigated. Even though these hybrid TCEs have been investigated for applications in various electronic devices including solar cells, organic light emitting diodes, flexible heaters, flexible sensors, and touch panels [14,15,16,17,18], no extensive studies have been reported on the application of hybrid electrodes for PDLC-based smart windows.

In this study, the hybrid Ag NW–GO electrode was used as a substitute for an ITO electrode in a PDLC. To the best of our knowledge, we report for the first time Ag NW–GO hybrid electrodes integrated into smart windows based on PDLCs. Because of the inverse relationship between optical transmittance and electrical resistivity of TCE, the optical properties of TCE deteriorate with increasing electrical conductivity. However, Ag NW–GO hybrid structure can reduce the resistances of Ag NW networks without affecting their transmittances. In this case, we have described the preparation of highly conductive and transparent Ag NW–GO hybrid electrodes by a simple spray-coating technique for the fast production of large-area flexible smart windows with a decreased production cost.

## 2. Materials and Methods

### 2.1. Ag NW–GO Hybrid Electrode Fabrication

A hybrid electrode was fabricated by using Ag NWs (Nanopyxis) dispersed in isopropyl alcohol (IPA) and GO (Uninanotech) dispersed in IPA. In order to fabricate the GO suspension, a 6.2 g/L GO suspension was diluted with IPA to 0.2 g/L and sonicated for 20 min. The polyethylene terephthalate (PET) substrates were cleaned with acetone, IPA, and deionized water. After drying, the PET films were treated with ultraviolet-ozone (UVO) for 5 min. Immediately after the UVO treatment, the Ag NW suspension was uniformly spray coated and annealed at 65 °C for 1 min. Lastly, in order to form Ag NW–GO hybrid electrodes, the GO suspension was spray coated onto the Ag NWs and dried at 65 °C for 5 min. For spray deposition, a fully automated spray coater was utilized.

### 2.2. PDLC Fabrication

The PDLCs, which is commercially available from Qingdao Liquid Crystal, were mixed with spacers at a weight ratio of 100:1 and stirred for 4 h to obtain a uniform thickness of the PDLC layer. The fully mixed solution was drop-dispersed on the substrate coated with Ag NW–GO. Subsequently, it was covered with another Ag NW–GO-coated substrate, which produced a sandwich structure (Figure S1). Lastly, the PDLC layer was photo-polymerized by using a mask aligner for 5 min.

## 3. Results and Discussion

**Figure 1:**
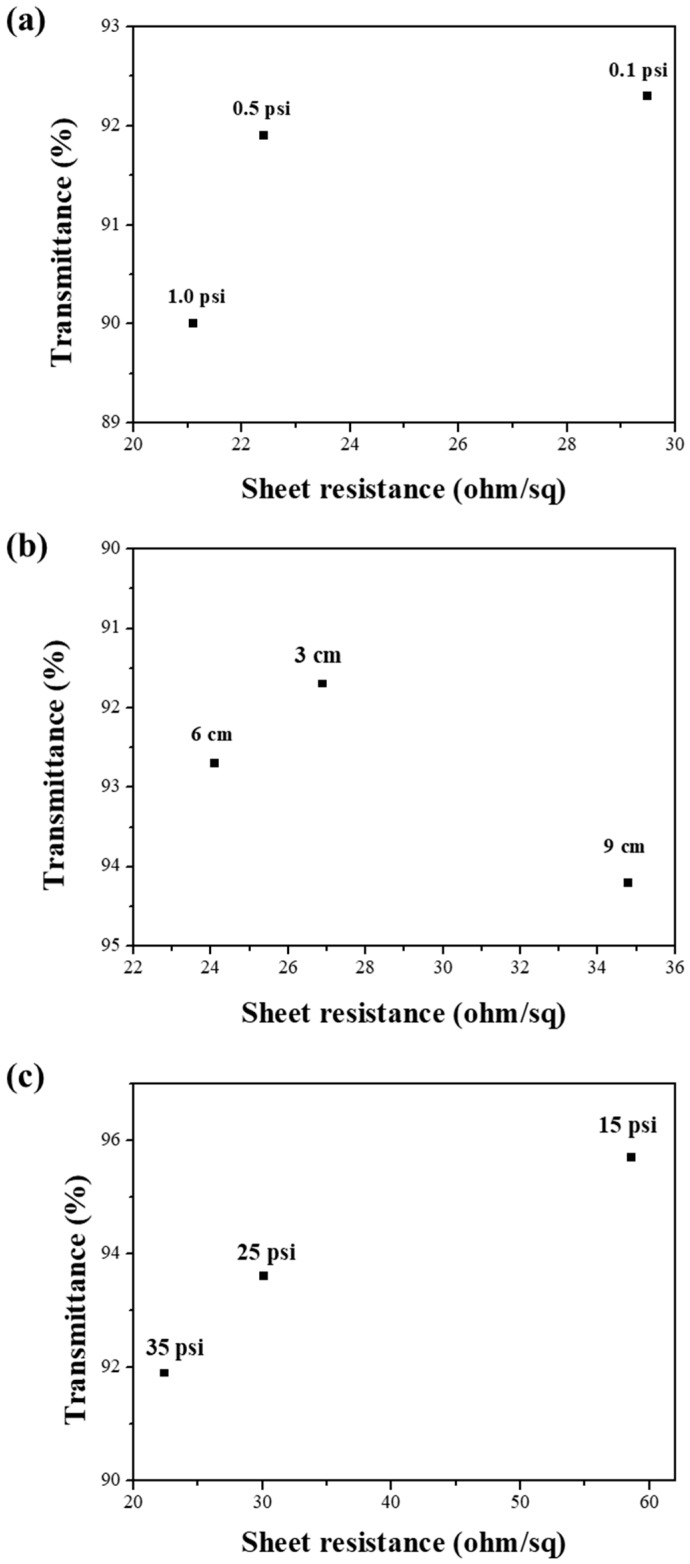
Transmittances at 550 nm and sheet resistances of the spray-coated Ag NW films as a function of the (**a**) dispensing pressure, (**b**) nozzle–to–substrate distance, and (**c**) nozzle pressure.

**Figure 2:**
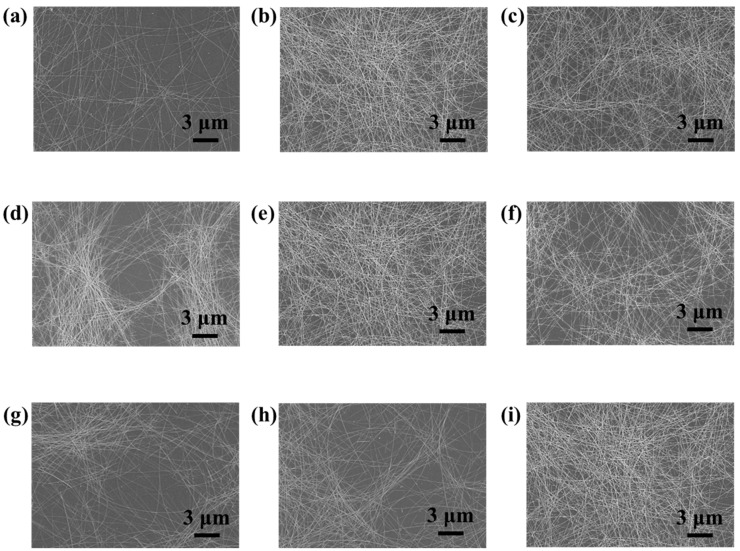
SEM images of Ag NW networks obtained with different spray-coating parameters including dispensing pressures of (**a**) 0.1 psi, (**b**) 0.5 psi, and (**c**) 1 psi, nozzle–to–substrate distances of (**d**) 3 cm, (**e**) 6 cm, and (**f**) 9 cm, and nozzle pressures of (**g**) 15 psi, (**h**) 25 psi, and (**i**) 35 psi.

In order to produce highly uniform, transparent, and conductive Ag NW networks by spray coating, various processing parameters such as dispensing pressure, spray pressure, nozzle–to–sample distance, scan speed, and substrate temperature should be simultaneously controlled [19]. We investigated the influence of each of these parameters on the morphology of the Ag NW network, which is known to affect their electro-optical properties. First, Ag NW networks were prepared at three different dispensing pressures: 0.1 psi, 0.5 psi, and 1 psi. Their optical transmittances (*T*) and sheet resistances (*R_s_*) were measured and their morphologies were characterized by using scanning electron microscopy (SEM). Figure 1a shows a decrease in the *T* and *R_s_* values with the increasing dispensing pressure. A higher dispensing pressure led to a higher flow rate of the Ag NW suspension, which affected the density of the Ag NW networks. As shown in Figure 2a,b and Figure S2a,b, the Ag NW density increased with the dispensing pressure. However, a high dispensing pressure could clog the nozzle, which leads to an irregular flow of the Ag NW suspension through the nozzle and, thus, to an irregular coating of Ag NWs. Ag NW-deficient areas were observed in Figure S2c due to the irregular deposition of Ag NWs at a dispensing pressure of 1 psi. In order to avoid this phenomenon, the dispensing pressure was maintained at 0.5 psi. Second, the nozzle–to–sample distance, which controls the mass of Ag NWs sprayed per unit area, was optimized to obtain uniform Ag NW networks [20]. The *T* and *R_s_* values in Figure 1b show that the density of Ag NWs increased with a decreasing nozzle–to–substrate distance. The nozzle–to–substrate distance was chosen to be 6 cm since a smaller distance led to nonuniform Ag NW networks while a larger distance led to low-density Ag NW networks, which is shown in Figure 2d–f and Figure S2d–f. Lastly, we investigated the effect of the nozzle pressure on the electro-optical performance of the Ag NW network. The nozzle pressure was chosen to be as high as 35 psi because an increased nozzle pressure increased the Ag NW density, as shown in Figure 2g–i. The relationship between *T* and *R_s_* of the Ag NW networks (Figure 1c) was consistent with the SEM results. A higher nozzle pressure led to large shear forces, which promoted the Ag NW suspension into smaller droplets that were beneficial for the deposition of more uniform films since small droplets tend to produce less prominent “coffee-staining” effects [21].

**Figure 3:**
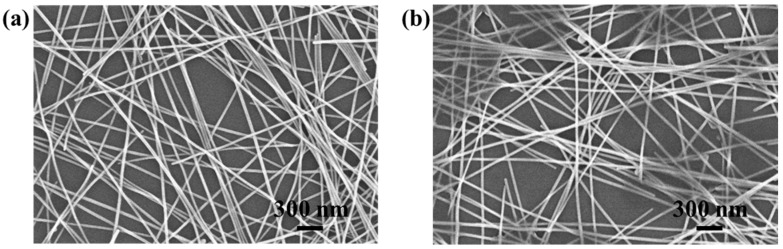
SEM images of the spray-coated (**a**) Ag NW network and (**b**) Ag NW network covered by GO nanosheets.

In order to enhance the electro-optical properties of the Ag NW networks, they were spray-coated with the GO suspension. Electrical conduction in an Ag NW network is dominated by the resistances at junctions of Ag NWs due to the percolative nature of conduction. The GO tends to bond with Ag NWs due to the strong electrostatic adhesion caused by the large number of oxygen-containing groups in GO [22]. The GO sheets adhered and wrapped around the Ag NWs, which led to the soldering of the inter-nanowire junctions and caused a significant reduction in the contact resistance of these junctions [23]. The optimized spray-coating parameters for the formation of Ag NW networks were also used for the spray coating of the GO suspension. As shown in Figure 3b and Figure S3, the Ag NW junctions were wrapped by the GO sheets and a small number of GO sheets were deposited in the optical pathway and unblocked by Ag NWs, which enhanced the electro-optical properties of the Ag NW–GO hybrid networks by reducing *R_s_* while minimizing the loss of *T*. The transmittances, sheet resistances, and figures of merit (FoMs) of the Ag NW and Ag NW–GO hybrid networks are summarized in Table 1. The spray-coated Ag NW–GO hybrid networks exhibited excellent properties. A representative film had *T* = 90.7% and *R_s_* = 15.6 Ω/sq. Table 1 shows that the FoM was enhanced upon the formation of the Ag NW–GO hybrid network when compared to that of the Ag NW network.

**Figure 4:**
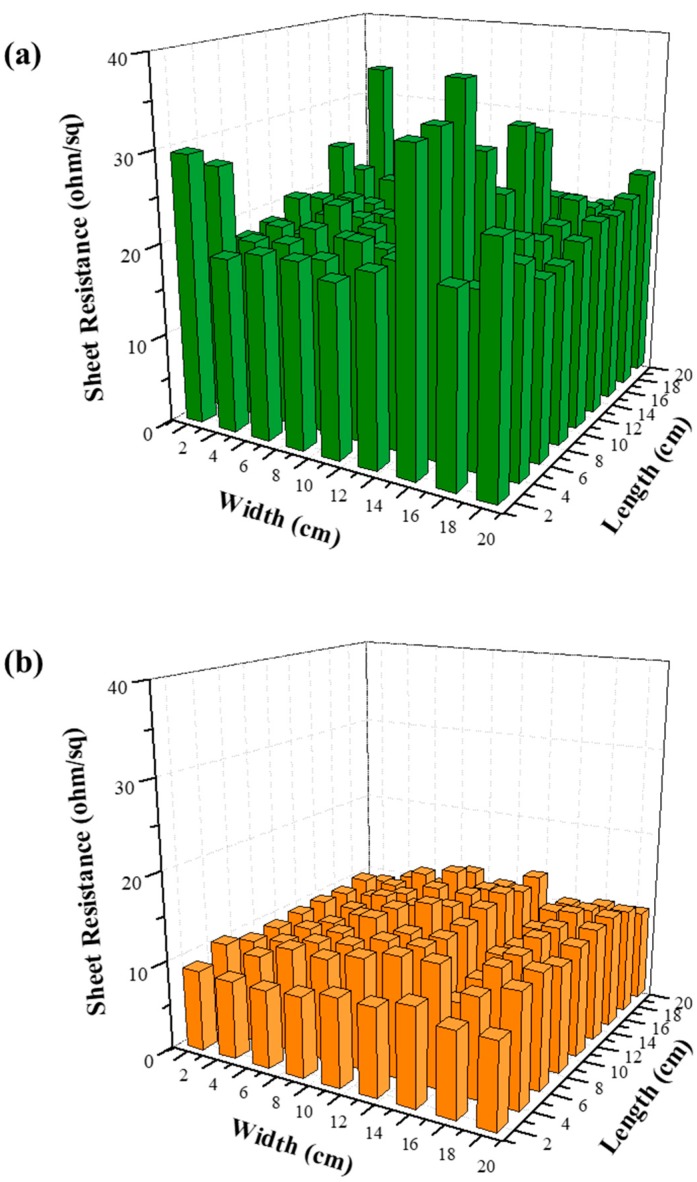
Histograms of the sheet resistances of the large-area, (**a**) Ag NW, and (**b**) Ag NW–GO hybrid electrodes.

Furthermore, the uniformity of *R_s_* of the Ag NW networks, which is one of the most important quality factors for large-area transparent conductive films, was improved upon the formation of the Ag NW–GO hybrid networks. The uniformities of large-area Ag NW and Ag NW–GO networks (200 mm × 200 mm) were estimated by measuring their *R_s_* values at 81 points with intervals of 20 mm in the horizontal and vertical directions. Figure 4 shows the distribution of the *R_s_* values of the Ag NW and Ag NW–GO networks. The standard deviation of *R_s_* of the Ag NW and Ag NW–GO networks were 4.21 Ω/sq and 1.48 Ω/sq, respectively. Therefore, the improved electro-optical properties and high uniformity of the Ag NW–GO network fabricated by spray coating made it suitable for large-area smart window applications.

**Figure 5:**
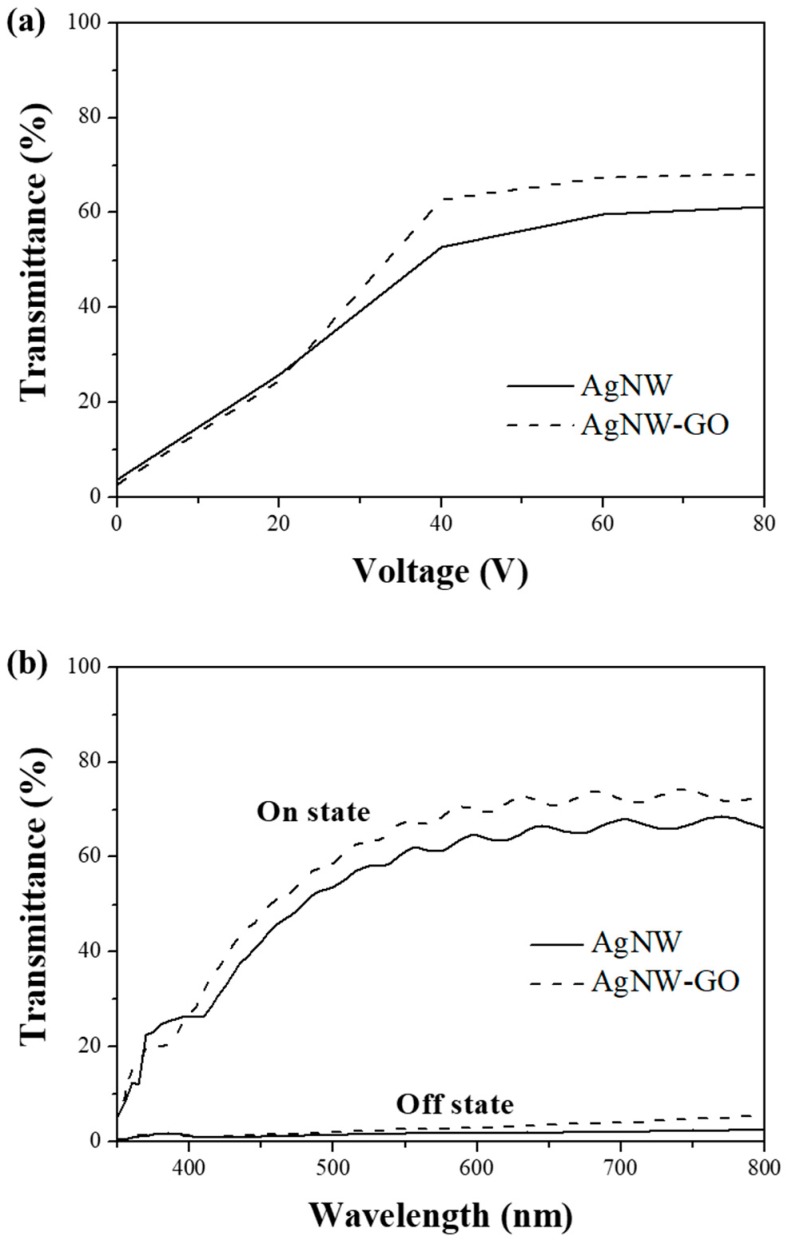
(**a**) Transmittances of the Ag NW and Ag NW–GO PDLCs under applied voltages in the range of 0 to 80 V at a wavelength of 550 nm. (**b**) Transmittance spectra of the Ag NW and Ag NW–GO PDLCs in the on- (80 V) and off- (0 V) states.

The transmittances of the PDLC were measured at applied voltage amplitudes in the range of 0 to 80 V. Voltage–transmittance characteristics of PDLCs fabricated with Ag NW and Ag NW–GO hybrid electrodes are shown in Figure 5a. The difference in driving voltage between these two sets of PDLCs was clear. The driving voltage of the PDLC with Ag NW electrodes was larger than that of the PDLC fabricated by using Ag NW–GO electrodes. The transmittance of the PDLC based on the Ag NW–GO hybrid electrodes could reach 63% at 40 V while that of the Ag NW-based PDLC was only 53% at 40 V and the maximum transmittance was only 61% at 80 V. This result implied that the lower *R_s_* of the Ag NW–GO electrodes than that of the Ag NW electrodes led to a lower driving voltage and lower energy consumption. Figure 5b shows the transmittances of the Ag NW and Ag NW–GO-based PDLCs in the on- (80 V) and off- (0 V) states as a function of the wavelength. The transmittance differences at 550 nm between the on-states and off-states for the Ag NW and Ag NW–GO-based PDLCs were 57% and 65%, respectively (Figure S4). Therefore, the Ag NW–GO-based PDLC was more desirable for use as a smart window since it was not only more transparent in the on state but could also be operated at a lower voltage.

**Figure 6:**
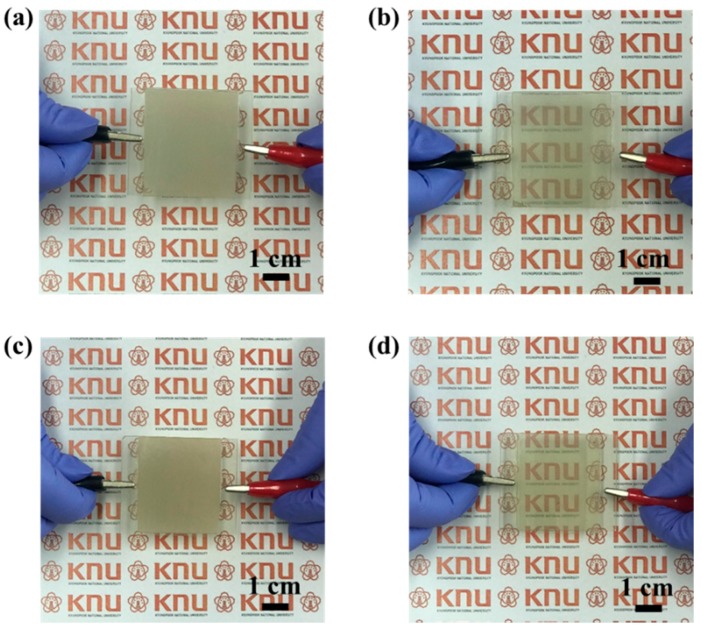
Photographs of the Ag NW PDLC in the (**a**) off-states and (**b**) on-states. Photographs of the Ag NW–GO PDLC in the (**c**) off-states and (**d**) on-states.

Photographs of the fabricated PDLCs in the on-states and off-states are shown in Figure 6. The difference in transmittance between the on-states and off-states was evident and in agreement with the results in Figure 5b. The fabricated PDLC was opaque in the off-state at which the printed text below the PDLC could hardly be observed. On the contrary, in the on-state, the printed text underneath the PDLC could be clearly observed since the PDLC was transparent. In the case of the Ag NW–GO-based PDLC, the printed text appears clearer than in the case of the Ag NW-based PDLC due to its high transmittance in the on-state. The high contrast between the on-states and off-states made the PDLC with Ag NW–GO suitable for smart windows.

**Figure 7:**
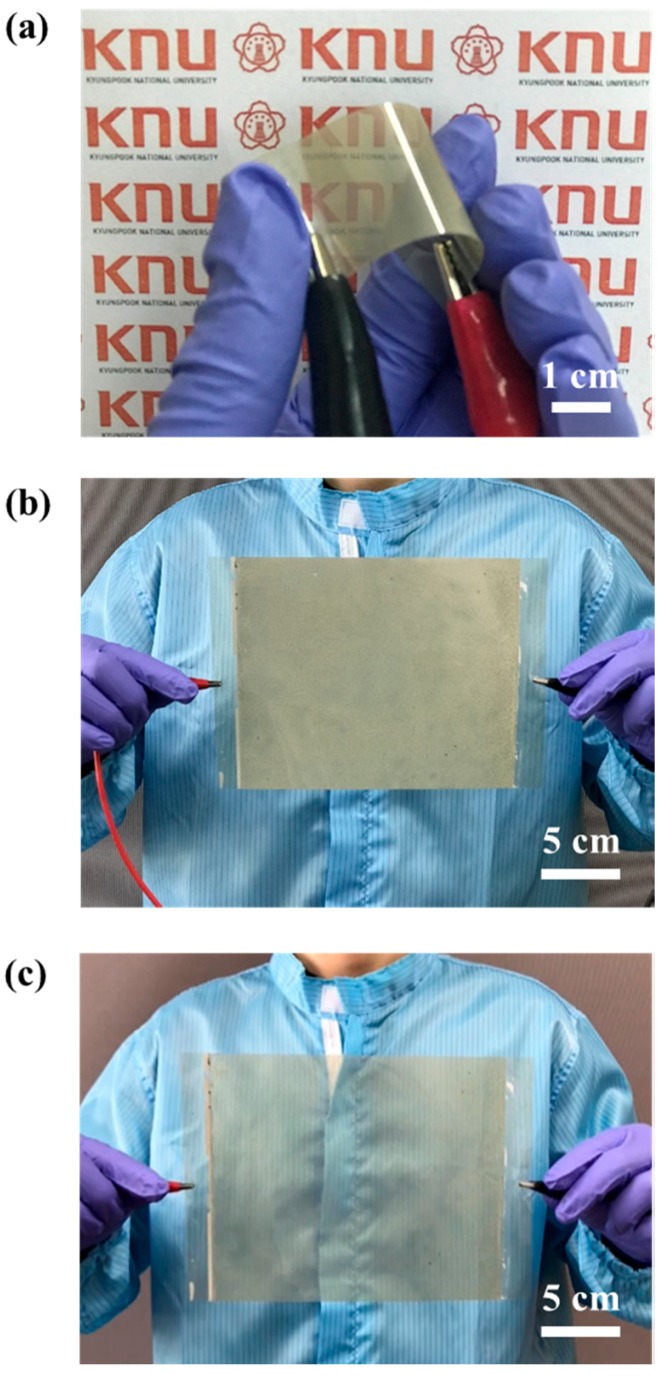
(**a**) Photograph of the flexible PDLC in the on-state. Photographs of the large-area PDLC in the (**b**) off-states and (**c**) on-states.

Lastly, we aimed to fabricate a flexible large-area PDLC by utilizing the advantage of the PDLC based on the Ag NW–GO electrodes that were obtained by using the spray-coating technique. In order to fabricate a flexible large-area PDLC, Ag NW–GO was spray-coated on a PET substrate rather than on a glass substrate [24,25,26]. As shown in Figure 7a, the flexible PDLC operated perfectly and no damage was observed even though the PDLC was bent. The images of the large-area PDLC with the Ag NW–GO electrodes in Figure 7b,c show that the large-area PDLC operated uniformly in both on-states and off-states. This implied that the electric field between the Ag NW–GO electrodes was sufficiently uniform, which is consistent with the results illustrated in Figure 4b.

## 4. Conclusions

The spray-coating conditions were optimized for the fabrication of Ag NW–GO hybrid TCEs with high *T* and low *R_s_* values, which can be used as superior alternatives to ITO TCEs. The PDLCs based on the Ag NW–GO electrodes exhibited a high transmittance of 63% in the on-state under a low driving voltage (40 V) due to the low *R_s_*. The new class of flexible large-area PDLCs presented in this study demonstrate the promising potential of the spray-coated Ag NW–GO hybrid electrodes for PDLCs. This study provides a significant advancement toward the realization of flexible smart windows for future flexible optoelectronic applications.

## Figures and Tables

**Table 1:** Sheet resistances, transmittances, and FoMs of the Ag NW and Ag NW–GO films.

Electrode	Sheet Resistance (Ω/sq)	Transmittance (%) (at 550 nm)	FoM (10^−3^ Ω^−1^)
Ag NW	22.8	92.0	19.1
Ag NW–GO	15.6	90.7	24.2

## Supplementary Materials

The following are available online at http://www.mdpi.com/1996-1944/11/11/2231/s1, Figure S1: Schematic illustration of the steps involved in fabricating PDLC, Figure S2: SEM images of Ag NW networks obtained with different spray-coating parameters, including dispensing pressures of (a) 0.1, (b) 0.5, and (c) 1 psi, nozzle–to–substrate distances of (d) 3, (e) 6, and (f) 9 cm, and nozzle pressures of (g) 15, (h) 25, and (i) 35 psi., Figure S3: The Raman spectra of Ag NW–GO, Figure S4: Transmittance spectra of the ITO, Ag NW and Ag NW–GO PDLCs in the on-state (80 V) and off-state (0 V).

## References

[1] Lampert C.M. (2003). Large-area smart glass and integrated photovoltaics. Sol. Energ. Mater. Sol. Cells.

[2] Sol J.A., Timmermans G.H., van Breugel A.J., Schenning A.P., Debije M.G. (2018). Multistate Luminescent Solar Concentrator “Smart” Windows. Adv. Energy Mater..

[3] Granqvist C.G. (2014). Electrochromics for smart windows: Oxide-based thin films and devices. Thin Solid Films.

[4] Cupelli D., Nicoletta F.P., Manfredi S., Vivacqua M., Formoso P., De Filpo G., Chidichimo G. (2009). Self-adjusting smart windows based on polymer-dispersed liquid crystals. Sol. Energ. Mater. Sol. Cells.

[5] Kim Y., Jung D., Jeong S., Kim K., Choi W., Seo Y. (2015). Optical properties and optimized conditions for polymer dispersed liquid crystal containing UV curable polymer and nematic liquid crystal. Curr. Appl. Phys..

[6] Kim Y., Kim K., Kim K.B., Park J., Lee N., Seo Y. (2016). Flexible polymer dispersed liquid crystal film with graphene transparent electrodes. Curr. Appl. Phys..

[7] Khaligh H.H., Liew K., Han Y., Abukhdeir N.M., Goldthorpe I.A. (2015). Silver nanowire transparent electrodes for liquid crystal-based smart windows. Sol. Energ. Mater. Sol. Cells.

[8] Chou T., Chen S., Chiang Y., Chang T., Lin C., Chao C. (2017). Highly conductive PEDOT: PSS film by doping p-toluenesulfonic acid and post-treatment with dimethyl sulfoxide for ITO-free polymer dispersed liquid crystal device. Org. Electron..

[9] Lim J., Lee S., Kim S., Kim T., Koo H., Kim H. (2017). Brush-paintable and highly stretchable Ag nanowire and PEDOT: PSS hybrid electrodes. Sci. Rep..

[10] Ricciardulli A.G., Yang S., Wetzelaer G.A., Feng X., Blom P.W. (2018). Hybrid Silver Nanowire and Graphene-Based Solution-Processed Transparent Electrode for Organic Optoelectronics. Adv. Funct. Mater..

[11] Chen D., Liang J., Liu C., Saldanha G., Zhao F., Tong K., Liu J., Pei Q. (2015). Thermally stable silver nanowire–polyimide transparent electrode based on atomic layer deposition of zinc oxide on silver nanowires. Adv. Funct. Mater..

[12] Kim C., Jung C., Oh Y., Kim D. (2017). A highly flexible transparent conductive electrode based on nanomaterials. NPG Asia Mater..

[13] Jang J., Im H., Jin J., Lee J., Lee J., Bae B. (2016). A Flexible and Robust Transparent Conducting Electrode Platform Using an Electroplated Silver Grid/Surface-Embedded Silver Nanowire Hybrid Structure. ACS Appl. Mater. Interfaces.

[14] Zhang Q., Di Y., Huard C.M., Guo L.J., Wei J., Guo J. (2015). Highly stable and stretchable graphene–polymer processed silver nanowires hybrid electrodes for flexible displays. J. Mater. Chem. C.

[15] Lee J., Lee P., Lee H.B., Hong S., Lee I., Yeo J., Lee S.S., Kim T., Lee D., Ko S.H. (2013). Room-temperature nanosoldering of a very long metal nanowire network by conducting-polymer-assisted joining for a flexible touch-panel application. Adv. Funct. Mater..

[16] Zilberberg K., Gasse F., Pagui R., Polywka A., Behrendt A., Trost S., Heiderhoff R., Görrn P., Riedl T. (2014). Highly Robust Indium-Free Transparent Conductive Electrodes Based on Composites of Silver Nanowires and Conductive Metal Oxides. Adv. Funct. Mater..

[17] Kim D., Zhu L., Jeong D., Chun K., Bang Y., Kim S., Kim J., Oh S. (2013). Transparent flexible heater based on hybrid of carbon nanotubes and silver nanowires. Carbon.

[18] Fan Z., Liu B., Liu X., Li Z., Wang H., Yang S., Wang J. (2013). A flexible and disposable hybrid electrode based on Cu nanowires modified graphene transparent electrode for non-enzymatic glucose sensor. Electrochim. Acta.

[19] Lee J., Shin D., Park J. (2016). Fabrication of silver nanowire-based stretchable electrodes using spray coating. Thin Solid Films.

[20] Scardaci V., Coull R., Lyons P.E., Rickard D., Coleman J.N. (2011). Spray deposition of highly transparent, low-resistance networks of silver nanowires over large areas. Small.

[21] Deegan R.D., Bakajin O., Dupont T.F., Huber G., Nagel S.R., Witten T.A. (2000). Contact line deposits in an evaporating drop. Phys. Rev. E.

[22] Ha B., Jo S. (2017). Hybrid Ag nanowire transparent conductive electrodes with randomly oriented and grid-patterned Ag nanowire networks. Sci. Rep..

[23] Liang J., Li L., Tong K., Ren Z., Hu W., Niu X., Chen Y., Pei Q. (2014). Silver nanowire percolation network soldered with graphene oxide at room temperature and its application for fully stretchable polymer light-emitting diodes. ACS Nano.

[24] Singh A., Salmi Z., Joshi N., Jha P., Decorse P., Lecoq H., Lau-Truong S., Jouini M., Aswal D., Chehimi M. (2013). Electrochemical investigation of free-standing polypyrrole–silver nanocomposite films: A substrate free electrode material for supercapacitors. RCS Adv..

[25] Kim Y., Hong J., Lee S. (2006). Fabrication of a highly bendable LCD with an elastomer substrate by using a replica-molding method. J. Soc. Inf. Disp..

[26] Kim I., Kim T., Lee S., Kim B. (2018). Extremely Foldable and Highly Transparent Nanofiber-Based Electrodes for Liquid Crystal Smart Device. Sci. Rep..

