# Supplementary material for: Spray Deposition of Ag Nanowire–Graphene Oxide Hybrid Electrodes for Flexible Polymer–Dispersed Liquid Crystal Displays
Source: Materials (Basel). 2018 Nov 9;11(11):2231. doi: 10.3390/ma11112231 (PMC6266263; doi:10.3390/ma11112231)
Supplement: Supplementary file 1 [file materials-11-02231-s001.pdf]

Supplementary Materials

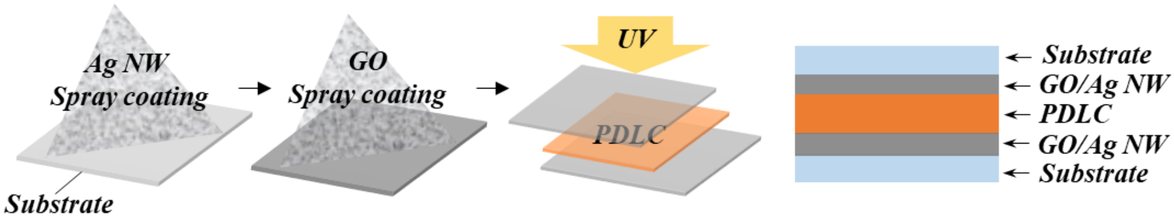

Figure S1. Schematic illustration of the steps involved in fabricating PDLC.

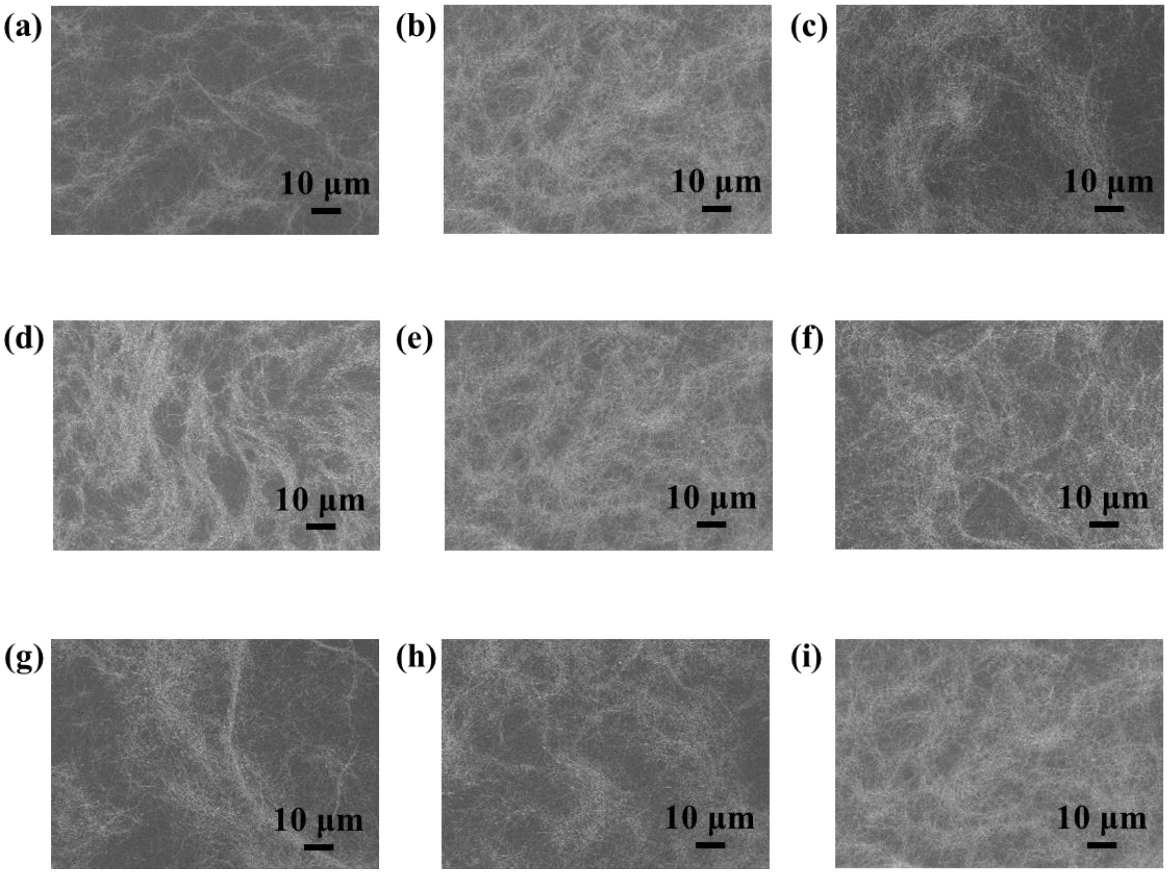

Figure S2. SEM images of Ag NW networks obtained with different spray-coating parameters, including dispensing pressures of (a) 0.1, (b) 0.5, and (c) 1 psi, nozzle–to–substrate distances of (d) 3, (e) 6, and (f) 9 cm, and nozzle pressures of (g) 15, (h) 25, and (i) 35 psi.

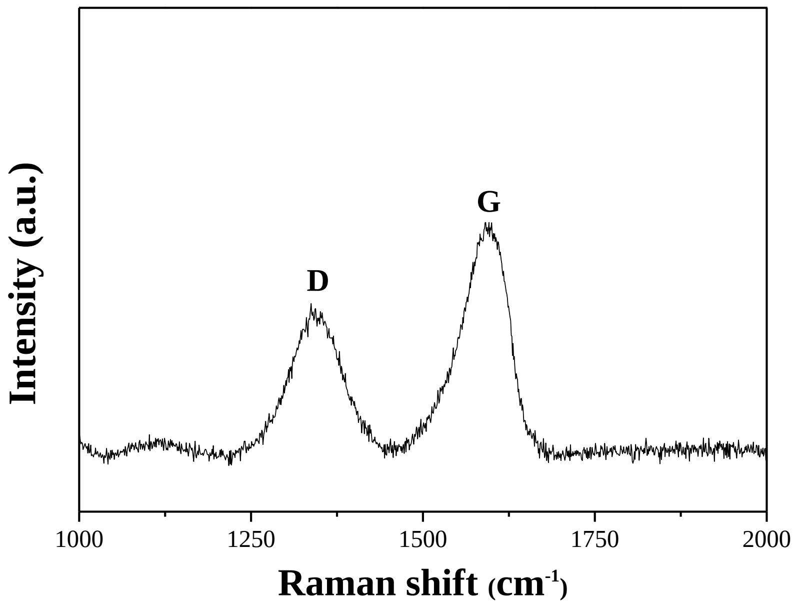

Figure S3. The Raman spectra of Ag NW–GO.

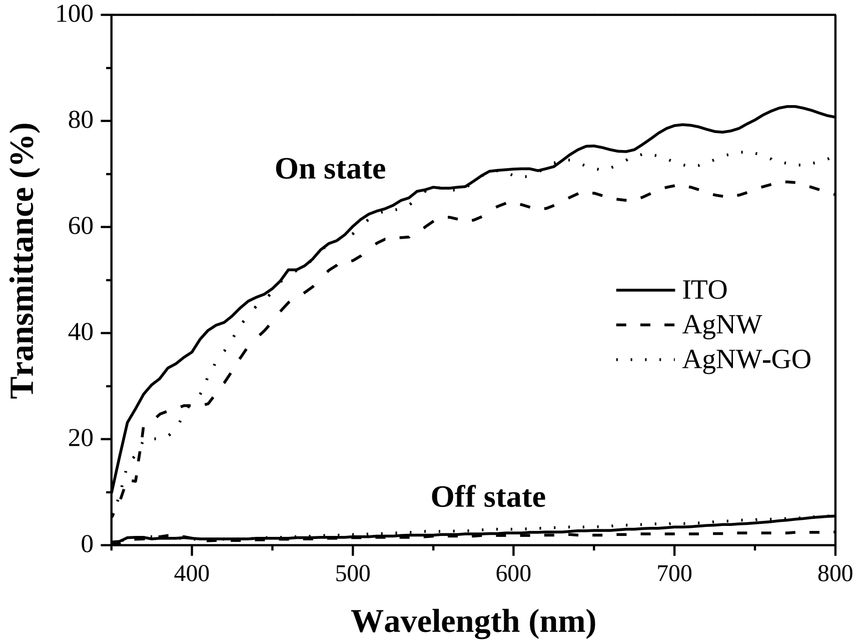

Figure S4. Transmittance spectra of the ITO, Ag NW and Ag NW–GO PDLCs in the on-state (80 V) and off-state (0 V).
